# Anatomy and Physiology

**Published:** 1876-02

**Authors:** 


### Summary of Progress in the Medical Sciences.

### I. Anatomy and Physiology.

1. *Congenital Myo-Sarcoma of the Kidneys.* J. Cohnheim. (*Virch. Arch.* LXV; *Centralb. f. chir.*, 1875, No. 51.)

A girl, aged one year, died of a large tumor which filled the abdominal cavity almost entirely, crowding the intestines to the right side, and the diaphragm upward, and completely compressing the bladder. The post-mortem examination showed this tumor to have grown out of the substance of the left kidney. The larger half of this kidney still existed in the shape of a small, smooth appendix, clinging to the tumor. This was composed of a great number of smaller roundish tumors, and one such tumor was also contained in the right kidney, which otherwise was of a normal size. The tumors resembled the large fibrous tumors of the uterus, as well as medullary osteo-sarcoma. But the microscope showed that the larger portion was built up by *striped muscular fibres;* in a few smaller lumps only, the structure of the round-cell sarcoma was found.

The case is very interesting, on account of the size and large mass of the muscular growth, and because a neoplasma, mainly composed of striped muscular fibres, has never before been found in the kidneys. From the fact that both kidneys showed the same pathological condition, the morbid process may be considered as a "vitium primæ formationis."

2. *The Lymphatic Vessels of the Joints.* H. Tillmanns. (*Centralb. f. chir.*, 1875, No. 51.)

The anatomical distribution of the lymphatics, in the synovial membranes, has lately been made the object of histological studies by Tillmanns, in Leipzig. In order to inject these vessels, he filled the joint with a colored fluid, and tried, by alternate flexions and extensions, to force it into the lymphatics of the synovial membrane. Failing with this method, he injected a solution of nitrate of silver (half per cent.) into the tissues of the capsule by the hypodermic syringe. By this method he obtained very nicely injected preparations, and ascertained the following facts: The superficial lymphatic vessels are situated directly under the epithelium of the synovial membrane, but they have no open communications (stomata) with the cavity of the joint, like the lymphatics of other serous membranes. The finest capillaries of the blood vessels do not seldom crowd in between the epithelium and the lymphatics. Further on in the tissues, numerous and larger lymphatics are seen to wind around the blood vessels. The lymphatics of the capsule could never be traced into the osseous tissue, nor could any be discovered in the cartilage.

3. *A New Sphygmograph.* A. T. Keyt, M.D. (*N. Y. Med. Jour.*, Jan., 1876.)

This instrument, which the author claims embodies an original application and working out of a principle heretofore in use, resembles the sphygmographs previously in vogue, that of Marey most, but differs in the means of receiving and using the force of the pulse. While the older instruments made use of a metallic spring which was elevated at every pulse-beat, this one utilizes the elastic membrane and a liquid to "receive and transmit to the tracing lever the movements of the pulsating artery." There is a tube, with an expanded base, across the open end of which an elastic membrane is stretched; the other end of the tube is also expanded slightly, and has another elastic membrane covering its orifice. This tube being full of fluid, the membrane first described is pressed firmly against the artery—of course the membrane at the other end of the tube, through the medium of the fluid, pulsates with the artery. The broad, flattened head of a pin, rests upon this membrane, and rising and falling with every oscillation of the membrane, its impulses, by a simple contrivance, are transmitted to a tracing lever, and so the sphygmographic workings are registered. The tracings are made upon the surface of smoked glass, which is propelled against the tracing lever by means of clockwork, such as is used in Marey's sphygmograph. The instrument, as described, is ingeniously constructed, and has a number of mechanical devices for regulating, to a nicety, the working of the machine. The straight, vertical glass tube, with the expanded base and membrane, the tube graduated in inches and fractions, and filled with water, Dr. K. uses alone for the purpose of presenting to the eye, in the oscillations of the fluid, a representation of the pulse-beat. This he names the sphygmometer, and it forms a part of the sphygmograph, so that, by the simple turning of a thumb-screw, the instrument may be used as either a sphygmometer or a sphygmograph. By placing the bulb of the instrument over the apex of the heart—the patient being in the supine position—it is either a cardiograph or cardiometer at will.

The author thus summarizes the advantages of his invention: "The new sphygmograph is a very convenient instrument. It is light in weight, small in bulk, portable. It need not easily get out of order; the membranes even, if taken care of, will maintain their integrity for a considerable time, and when at last these show failure, they can be readily replaced by new ones. It can be easily placed and retained in position. It needs no strapping to hold it in position." "It is adapted to trace the pulsations of any of the superficial arteries; and, as a cardiograph, to trace the heart's pulsations with the same facility that it traces the arterial." "Such is the facility of its employment, that it entails upon the very feeble no uneasiness or annoyance, and the pulses of infants can, without trouble, be recorded by it."

Quite a number of tracings are shown, made on the same artery at the same sitting, with the new and Marey's instruments respectively, which, it must be said, give a very creditable exhibition of the capacity of the former.

4. *Physiological action of Lobelina.* OTT. (*Phila. Med. Times*, Dec. 11, 1875.)

Dr. Isaac Ott, Demonstrator of Physiology, University of Pennsylvania, has again favored the medical world with another admirable therapeutic study. This time he investigated the active principle of lobelia, and he gives the following conclusions as to its physiological action :

1. Lobelina, like nicotina and conia, paralyzes the motor nerves.

2. Lobelina does not destroy the functions of the sensory nerves or the striated muscles.

3. Lobelina, nicotina, and conia, depress the excitability of the spinal cord.

4. Lobelina destroys voluntary movement and co-ordinating power.

5. It temporarily decreases the pulse, followed by a subsequent increase, often beyond normal. Nicotina does the same. Lautenbach states that conia increases the heart-beat.

6. This action on the pulse is due to an action on the cardio-motor ganglia, provided that atropin paralyzes the cardio-inhibitory ganglia.

7. Lobelina, nicotina, and conia, temporarily decrease the pressure, followed by a rise much beyond normal.

8. This increase of pressure is due either to a peripheral vaso-motor action, or to an excitation of the spinal vaso-motor centres.

9. Lobelina, nicotina, and conia, paralyze the pneumogastrics.

10. In large doses it paralyzes the vaso-motor centre, both to direct and to indirect irritation.

11. Lobelina, nicotina, and conia, accelerate the respiratory movements.

12. After section of the vagi, lobelina and conia cause no increase of respiratory movements.

13. It increases and then decreases the temperature; nicotina and conia decrease the temperature.

